# A Novel Case of Weissella confusa Infective Endocarditis of a Bio-Prosthetic Valve: Management and Treatment

**DOI:** 10.7759/cureus.37514

**Published:** 2023-04-13

**Authors:** Saleh A Massasati, Saba Waseem

**Affiliations:** 1 Internal Medicine, Conemaugh Memorial Medical Center, Johnstown, USA

**Keywords:** mortality, rare organism, vancomycin resistance, bacteremia in immunocompetent, weisella confusa, bio-prosthetic valve, infective endocarditis

## Abstract

Weissella confusa is a rare gram-positive, non-spore-forming, catalase-negative, gram-positive coccobacillus, and a pleomorphic gram-positive rod (GPR) often misidentified as Lactobacillus genus. It was first discovered in 1993 and is becoming identified due to the increasing use of DNA sequencing. The true incidence of this species has likely been underestimated and it has been implicated in poly-microbial bacteremia. We present an exceedingly rare case of its presentation found incidentally in a patient with a bio-prosthetic aortic and mitral valve that was successfully managed and treated.

## Introduction

The Weissella genus is a gram-positive bacteria that is a non-spore-forming, hetero-fermentative, facultative anaerobe, that is catalase-negative, and alpha-hemolytic that appears as short rods or coccobacillus in pairs and chains [1]. Based on their unusual gram stain morphology and inherent resistance to vancomycin, it has been confused with Lactobacillus especially when identified using commercial kits [1-3].

Identification of Weissella is challenging. Weissella was originally identified as a unique genus in 1993 based on 16S rRNA gene sequence analysis. Weissella constitutes a distinct phylogenetic group and is separate from other lactic acid bacteria. To date, 19 species have been identified. It is often misidentified as Lactobacillus-like or Viridans streptococci and accurate identification is not possible by traditional or commercial phenotypic identification methods. Therefore the gold standard to identify is molecular DNA sequencing involving 16S rRNA [1-3].

It is considered generally an opportunistic pathogen. Weissella confusa can grow at 25°C, 35°C, and 45°C and has been found in raw milk, feces, saliva, breast milk, urine, fermented cereals, and meat products. It is a common inhabitant of the human gastrointestinal system and vaginal microbiota. The true incidence of Weisella infection in humans is likely underestimated [1].

Weissella confusa bacteremia has been diagnosed as a poly-microbial infection in immunocompromised and immunocompetent patients. Cases range from post-operative osteomyelitis to intramural hematomas of the aorta, and patients on total parenteral nutrition [1].

## Case presentation

A 92-year-old female with a past medical history significant for a 19 mm Edwards Magna pericardial bovine aortic valve (AV) prosthetic implantation (Edwards Lifesciences, Irvine, CA) and a 25 mm porcine St. Jude bio-prosthetic mitral valve (MV) replacement (St. Jude Medical, Inc., St Paul, MN) in 2014, heart failure with persevered ejection fracture (HFpEF), paroxysmal atrial fibrillation on apixaban, chronic kidney disease (CKD) stage 3B, hypothyroidism, and prior lumbar fusion presented in the fall of 2021 due a two-week history of generalized weakness, dyspnea at rest, and intermittent dark stools.

Upon presentation to the emergency department, she was hemodynamically stable and afebrile. Initial labs were significant for a hemoglobin of 7.4 g/dL, hematocrit of 24% (baseline 12.9 g/dL /40%), platelet count of 106 10*3/uL, and B-type natriuretic peptide (BNP) of 295 pg/mL. Initial CT chest without contrast showed mild pulmonary edema, borderline cardiomegaly, and a severely calcified mitral valve annulus. Due to concerns of a gastrointestinal (GI) bleed, she was transfused with two units of packed red blood cells (PRBC) and admitted for further medical management.

Over the next 24 hours, the patient’s temperature trended upwards and peaked at 37.9°C. Initially, this was attributed to the blood transfusion which prompted two sets of peripheral aerobic and anaerobic blood cultures to be ordered. Both sets returned with high-grade Weisella confusa. Therefore the infectious disease service was consulted.

Given her history of bio-prosthetic valves, infective endocarditis needed to be ruled out. Therefore inflammatory markers and transesophageal echocardiography (TEE) were ordered, and the patient was started empirically on daptomycin 8 mg intravenously every 48 hours due to her significant chronic kidney disease.

The TTE resulted in mitral valve leaflets that were severely thickened with severe annular calcification and a tricuspid valve with mildly thickened leaflets. Repeat blood cultures grew Weissella confusa 48 hours later, confirming the diagnosis with a total of four positive sets of aerobic and anaerobic blood culture specimens. Therefore a trans-esophageal echo (TEE) was ordered.

**Figure 1:**
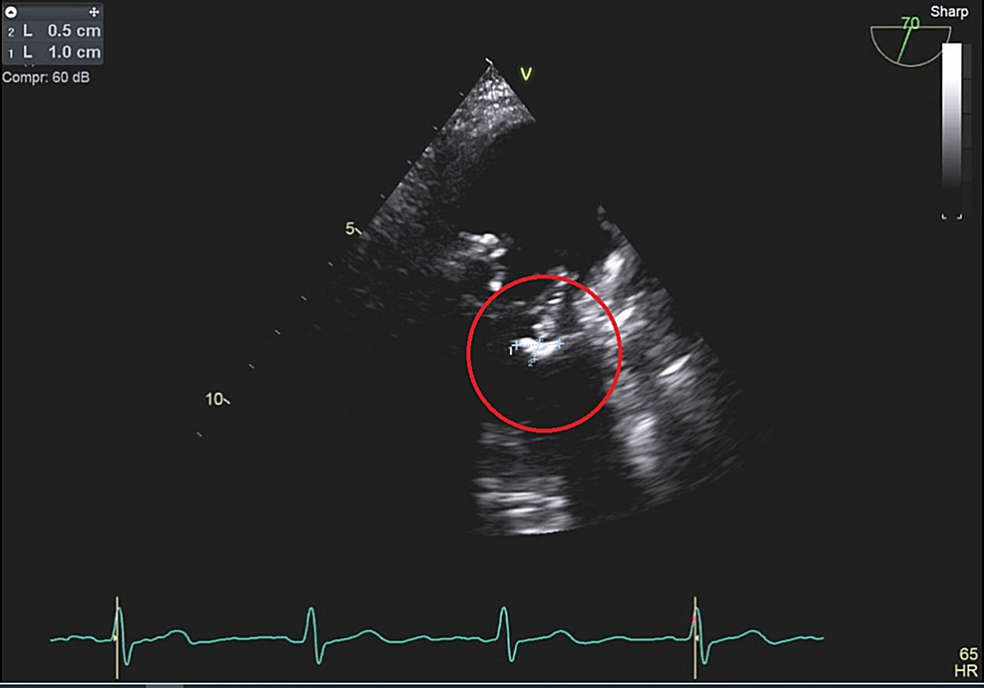
Transesophageal echocardiography (TEE) shows a bio-prosthetic mitral valve with a 1.0 x 0.5 cm vegetation on the posterior leaflet relative to the lateral wall of the left ventricle.

The TEE showed low normal systolic function with an ejection fraction of 50%-55%, a bio-prosthetic mitral valve with small vegetation on the posterior leaflet, and a 1.0 cm x 0.5 cm vegetation on the start of the mitral valve relative to the lateral wall of the left ventricle (Figure 1).

Afterward, cardiothoracic surgery was consulted and they deemed the patient as high risk for surgery due to her underlying co-morbidities and thus did not recommend surgery. In regards to her acute anemia, an upper endoscopy was carried out and a total of three angio-dysplastic lesions were clipped with stabilization of her anemia.

There was a total of 12 aerobic and anaerobic blood cultures that returned positive before a final set returned sterile for greater than 120 hours. Furthermore, there were improvements in inflammatory markers: pro-calcitonin 0.37 ng/mL to 0.34 ng/mL, C-reactive protein (CRP) 12.2 mg/dL to 5.6 mg/dL, and erythrocyte sedimentation rate (ESR) of 77 mm/hr to 52 mm/hr. The final recommendations per infectious disease were to continue daptomycin intravenously every 48 hours for a total of eight weeks with a repeat TEE to access response.

The repeat TEE 12 weeks afterward revealed a mildly decreased systolic function of 45%-50%. The mitral valve leaflets were mildly thickened, and the motion was normal with mild regurgitation and no stenosis. The tricuspid valve leaflets were mildly thickened with trace regurgitation, and no evidence of stenosis. Thus successful management and treatment of Weissella confusa bacteremia.​was achieved.

## Discussion

Infectious endocarditis is the inflammation of the endocardium that is primarily a disease caused overwhelmingly by gram-positive streptococci, staphylococci, and enterococci infection and the differential can vary greatly. Generally, symptoms are insidious and include fevers, chills, malaise, and fatigue that prompts medical evaluation within the first month. Predisposing risk factors include indwelling catheterization, intravenous drug use, recent pacemaker placement, or history of prosthetic valves predisposing to endocardial injury. Its treatment, prognosis, and management widely vary [4]. In this case, the patient presented had heart valves replaced predisposing her to an opportunistic infection.

Weissella confusa has been implicated in many poly-microbial and opportunistic infections. However, there have only been two documented cases of Weissella confusa endocarditis and subsequent bacteremia. Both of those cases involved native cardiac valves [5,6].

The antimicrobial susceptibilities of Weissella confusa are not fully understood. Weissella is known to be intrinsically resistant to vancomycin and has a high minimum inhibitory concentration (MIC) of ≥ 256 μg/ml. Many therapeutic agents including penicillin, daptomycin, piperacillin-tazobactam, and tigecycline have been used to treat Weissella confusa bacteremia successfully. Agents such as metronidazole, rifampin, teicoplanin, ceftazidime, and trimethoprim-sulphamethoxazole should be avoided [1,7].

## Conclusions

We present a rare and possible novel documented case of Weissella confusa endocarditis of a bio-prosthetic aortic and mitral valve that was diagnosed, managed, and treated successfully. The patient presented in this case was subsequently discharged in good health. We believe the primary source of this infection was likely GI in the setting of a recent esophageal gastric duodenoscopy. Follow-up trans-esophageal echo and blood cultures have remained negative for Weissella confusa.

## References

[1] Kamboj K, Vasquez A, Balada-Llasat JM (2015). Identification and significance of Weissella species infections. Front Microbiol.

[2] Lee MR, Huang YT, Liao CH, Lai CC, Lee PI, Hsueh PR (2011). Bacteraemia caused by Weissella confusa at a university hospital in Taiwan, 1997-2007. Clin Microbiol Infect.

[3] Shin JH, Kim DI, Kim HR, Kim DS, Kook JK, Lee JN (2007). Severe infective endocarditis of native valves caused by Weissella confusa detected incidentally on echocardiography. J Infect.

[4] Yallowitz AW, Decker LC (2022). Infectious Endocarditis. https://www.ncbi.nlm.nih.gov/books/NBK557641/.

[5] Lee W, Cho SM, Kim M, Ko YG, Yong D, Lee K (2013). Weissella confusa bacteremia in an immune-competent patient with underlying intramural hematomas of the aorta. Ann Lab Med.

[6] Flaherty JD, Levett PN, Dewhirst FE, Troe TE, Warren JR, Johnson S (2003). Fatal case of endocarditis due to Weissella confusa. J Clin Microbiol.

[7] Olano A, Chua J, Schroeder S, Minari A, La Salvia M, Hall G (2001). Weissella confusa (basonym: Lactobacillus confusus) bacteremia: a case report. J Clin Microbiol.

